# Lines of Evidence–Incremental Markings in Molar Enamel of Soay Sheep as Revealed by a Fluorochrome Labeling and Backscattered Electron Imaging Study

**DOI:** 10.1371/journal.pone.0074597

**Published:** 2013-09-06

**Authors:** Horst Kierdorf, Uwe Kierdorf, Kai Frölich, Carsten Witzel

**Affiliations:** 1 Department of Biology, University of Hildesheim, Hildesheim, Germany; 2 Tierpark Arche Warder e.V., Warder, Germany; Friedrich-Schiller-University Jena, Germany

## Abstract

We studied the structural characteristics and periodicities of regular incremental markings in sheep enamel using fluorochrome injections for vital labeling of forming enamel and backscattered electron imaging in the scanning electron microscope. Microscopic analysis of mandibular first molars revealed the presence of incremental markings with a daily periodicity (laminations) that indicated successive positions of the forming front of interprismatic enamel. In addition to the laminations, incremental markings with a sub-daily periodicity were discernible both in interprismatic enamel and in enamel prisms. Five sub-daily increments were present between two consecutive laminations. Backscattered electron imaging revealed that each sub-daily growth increment consisted of a broader and more highly mineralized band and a narrower and less mineralized band (line). The sub-daily markings in the prisms of sheep enamel morphologically resembled the (daily) prisms cross striations seen in primate enamel. Incremental markings with a supra-daily periodicity were not observed in sheep enamel. Based on the periodicity of the incremental markings, maximum mean daily apposition rates of 17.0 µm in buccal enamel and of 13.4 µm in lingual enamel were recorded. Enamel extension rates were also high, with maximum means of 180 µm/day and 217 µm/day in upper crown areas of buccal and lingual enamel, respectively. Values in more cervical crown portions were markedly lower. Our results are in accordance with previous findings in other ungulate species. Using the incremental markings present in primate enamel as a reference could result in a misinterpretation of the incremental markings in ungulate enamel. Thus, the sub-daily growth increments in the prisms of ungulate enamel might be mistaken as prism cross striations with a daily periodicity, and the laminations misidentified as striae of Retzius with a supra-daily periodicity. This would lead to a considerable overestimation of crown formation times in ungulate teeth.

## Introduction

Fully formed dental enamel is the hardest and most durable biological substance of the mammalian body [1]. Enamel is, therefore, normally the best preserved among the mineralized tissues in archaeological skeletal remains. Because enamel does not remodel or repair, the microstructure of enamel preserves information on the mode of the formative process, regarding both regular (physiological) growth events and pathological alterations caused by stress episodes [1]–[9]. The resulting growth marks in the enamel of both recent and ancient teeth enable a precise reconstruction of the timing of enamel formation and of stress episodes affecting enamel matrix formation, an information that is of major importance in the context of demographic, (palaeo-) pathological and forensic studies [1], [2], [7]–[9].

Enamel is produced by specialized cells of epithelial origin, the ameloblasts, and the temporal sequence of amelogenesis comprises the secretory and maturation stages [1], [10]. The newly secreted enamel matrix is soft and gel-like. It contains enamel-specific proteins (mostly amelogenins) that are essential for the proper growth of the thin enamel crystals that almost instantaneously form in the extracellular enamel matrix [11], [12]. During the secretory stage, the mineral content of the matrix increases to about 30% by weight. In the ensuing maturation stage, the enamel crystals expand in width and thickness as the extracellular matrix is removed, and the enamel achieves its final composition of about 96% mineral by weight [12], [13].

A fully active secretory ameloblast possesses a cellular extension (the Tomes’ process) at its distal pole with two sites of matrix secretion ([1], [12], [14], [15]. The first site constitutes a rim around the base (proximal portion) of the Tomes’ process, a region also called the ameloblast shoulder [5]. Here the matrix of the interprismatic enamel is secreted. The distal portion of the Tomes’ process (or the 'Tomes’ process proper' [15]) extends from the proximal portion and protrudes into a pit whose walls consist of interprismatic enamel. Matrix secretion occurs along one face of the distal portion of the Tomes’ process, and in this way the pit is filled with a prism, which is a long rod-like structure that extends from the enamel-dentin-junction (EDJ) towards the outer enamel surface (OES) [12], [14], [15].

At the onset of amelogenesis, a thin layer of initial enamel is secreted by ameloblasts that have not yet developed the distal portion of their Tomes’ processes and therefore possess only a single, flat secretory surface at their distal cell pole. As a consequence, all crystallites of the initially formed enamel show the same orientation and the enamel is aprismatic, i.e., does not show a differentiation into prisms and interprismatic enamel. Near the end of enamel matrix secretion, the distal portion of the Tomes’ process frequently regresses, so that again only a single flat secretory surface is present and a thin outer layer of aprismatic enamel is produced [12], [14], [15].

Ameloblasts undergo a rhythmic fluctuation in the rate of enamel matrix production. The rhythmically modulated secretory activity of the cells is reflected by regular incremental features that are visible in forming and mature enamel [1], [9], [11], [16], [17]. Numerous studies in primate (mostly human) enamel revealed that the microstructural growth marks in the enamel can be grouped into two basic categories: short period markings and long period markings. Short period markings have been associated with regular daily (circadian) changes in the rate of enamel matrix production [1]. In histological sections viewed in transmitted light, enamel prisms exhibit alternating bright and dark bands oriented perpendicular to their course. Most authors agree that these markings correspond to varicosities and constrictions along the prism long axis when viewed in the scanning electron microscope (SEM) [1], [9], [18]; however, the existence of a strict relationship between these two phenomena has been questioned by others [4], [19].

Vital labeling of forming enamel demonstrated that the number of cross striations, each consisting of a bright and a dark band, correlates with the time interval in days between the injections that produced these labels [20]–[23]. Also, the histological analysis of teeth from humans with a known age at death whose enamel was still forming when they died produced a very good match between the number of cross-striations in postnatal enamel and the postnatal age in days of the respective individuals [24]. Recently, it has been reported that circadian clock genes are expressed in mouse molars and it has been suggested that these genes are involved in the regulation of ameloblast and odontoblast functions, such as enamel and dentin matrix secretion and mineralization [25]. In line with this assumption, it was demonstrated that several clock proteins show circadian oscillatory expression patterns in ameloblasts [26] and that amelogenin gene expression in postnatal mouse molars oscillates with an approximately 24 h periodicity, with a significant 2-fold decrease during the dark period compared to the light period [11].

The regular long period markings of enamel are called (brown) striae of Retzius or Retzius lines. They denote successive positions of the forming front of the enamel. In lateral (imbricational) enamel, the striae of Retzius reach the OES and correspond to the perikyma grooves. In cuspal (appositional) enamel, the striae of Retzius are dome-shaped over the respective dentin horn and do not reach the OES [1], [9], [18], [23]. The ultimate physiological cause for the formation of striae of Retzius is unknown, but it has been hypothesized that their development may be triggered by the action of two independent oscillatory cycles that overlap at regular time intervals [23], [27]. The number of days between the formation of two consecutive striae of Retzius can be determined by counting the prism cross striations between them. This so called repeat interval can vary for different individuals of the same species, but has been shown to be constant for the teeth of a single individual [9], [18]. The stria of Retzius repeat interval in non-human primates ranges between 1 and 11 days and correlates with the body mass of the respective species, with smaller-bodied species having shorter repeat intervals than species with a larger body mass [17], [28]. In humans, the repeat interval is usually 8 or 9 days, but there is a considerable variation among individuals, with a typical range between 6 and 12 days [6], [17], [29].

A third type of incremental markings reported to occur in mammalian enamel are so called laminations (or laminated striations). Laminations were first described in aprismatic (prismless) surface enamel of human teeth [3], [30]–[32], and appear to be the most prominent incremental markings in the enamel of ungulate species [16], [33]–[36]. There is circumstantial and experimental evidence that laminations, like prism cross striations, constitute daily incremental markings [16], [33]–[36]. Laminations run a course parallel to that of the striae of Retzius, but with a much closer spacing, and denote successive daily positions of the enamel forming front during the secretory stage of amelogenesis [16], [23], [34]–[36]. The structural nature of laminations is debated. Smith [23] argues that they may result from the circadian rhythm manifesting across the entire enamel forming front, i.e. involving both, the prismatic and interprismatic growth regions. In contrast, Tafforeau et al. [16] consider laminations to represent three-dimensional alignments, and thus structural equivalents, of prism cross striations.

The activity of secretory ameloblasts can be impaired by a variety of stress factors that cause a disruption of normal enamel growth. Depending on the severity and duration of these growth disruptions, the affected teeth will exhibit irregular, i.e., accentuated and structurally altered incremental markings in their enamel and/or enamel hypoplasia, i.e., deficiencies in the amount of enamel formed [7]–[9], [18], [37]. If the periodicity of the regular incremental markings in the enamel is known, the position of these developmental defects within the enamel layer can be used to determine the timing of the stress events that caused their formation [7], [8], [38]–[40].

Based on the known periodicity of regular incremental enamel markings it is also possible to determine basic parameters of enamel growth [10], [17], [23], [41], [42]. Such parameters are: i) the enamel apposition rate (or daily secretion rate), i.e., the daily increase in the length of the enamel prisms along their course from the EDJ to the OES, ii) the duration of the appositional growth process, i.e., the number of days in which a single ameloblasts secrets matrix, and iii) the enamel extension rate, i.e., the rate at which new secretory ameloblasts differentiate from precursor cells along the presumptive EDJ from the tip of the dentin horn towards the future apical enamel border. Vital labeling of forming enamel with different chemicals (lead acetate, sodium fluoride and various fluorochromes) that leave a permanent trace in the enamel has been used to experimentally determine the abovementioned parameters in different species [22], [23], [34], [43]–[45]. Results obtained in various primate species demonstrated that in these taxa, prism cross striations reflect a daily periodicity of growth, while striae of Retzius typically reflect a longer (supra-daily) periodicity [22], [23], [44]. By contrast, the few experimental results obtained so far in ungulates (pig and deer) revealed the presence only of incremental markings with a daily periodicity [34], [43], [45]. It is, thus, questionable whether the framework developed to interpret the periodicity of incremental markings in primate enamel can without changes be applied also to ungulates. There is, therefore, a clear need for an experimental analysis of the periodicity of incremental markings in ungulate enamel.

Recent studies analyzed enamel hypoplasia in sheep as an indicator of periods of environmentally induced physiological stress with the aim to develop a model for interpreting the zooarchaeological record [36], [46]. Doing so requires a detailed knowledge of the timing of enamel formation in this species. The present paper reports the results of a fluorochrome labeling and backscattered electron imaging study that analyzed the pattern of enamel growth in sheep molars and the structural characteristics and periodicity of regular incremental markings in sheep enamel.

## Materials and Methods

The study was performed on mandibular first molars of six Soay sheep (*Ovis ammon* f. aries). We used Soay sheep because they are an unimproved breed whose development is more comparable to that of ancient sheep than that of modern breeds. This is of importance when the data on crown formation are used for comparison with sheep teeth from archaeological or prehistoric sites. Our experimental animals were taken from the Soay herd of the Tierpark Arche Warder e.V. (Warder, Germany), and were born between March 25^th^ and May 12^th^ 2009. The experiments were performed in accordance with the current animal care regulations in Germany, with permission of the responsible veterinary authorities of the federal state of Schleswig-Holstein (Ministerium für Landwirtschaft, Umwelt und ländliche Räume des Landes Schleswig Holstein; Az. V312-72241.123-34), and approval by the local animal ethics committee (Tierschutzkommission der Abteilung für Biologie der Universität Hildesheim). The experimental animals were earmarked individually and kept on pasture in an enclosure providing shelter huts and some additional food. From late October to April they were kept in a stable and given hay and pelleted feed.

Starting with the day of birth, the experimental animals received alternating intramuscular injections of calcein (Sigma Aldrich, product no. C0875, buffered to pH 7) at a dosage of 8 mg/kg body weight and oxytetracycline (ursocycline, Serumwerk Bernburg AG, product no. 09932159) at a dosage of 80 mg/kg body weight. Injections were performed between 9 and 12 a.m. at 14 day-intervals during three periods (Period 1: March 25^th^ to July 7^th^ 2009; Period 2: October 5^th^ to December 7^th^ 2009; Period 3: April 12^th^ to June 7^th^ 2010), with five injections given in each period (Table 1). However, three exceptions from the injection schedule occurred during the experiment. One animal (#79768) received its first injection not at the day of birth but at day six after birth. This animal was thus 6 days out of phase with the other individuals during the first injection period. In the second injection period, the interval between the second calcein injection and the second oxytetracycline injection was 21 days, not 14 days. In the third injection period, calcein was given as the first, third and fourth injection, while oxytetracycline was given as the second and fifth injection (Table 1). In the first injection period, the animals were treated individually at the respective postnatal age in days. In the second and third injection periods, all remaining animals were treated at the same day, causing some variation in postnatal age between the individuals at the respective injection dates. Age at death of the animals is given as days *post partum* (day of birth = day 0).

**Table 1:** Protocol of the dates of fluorochrome injections and the respective ages in postnatal days (0 =  day of birth) of the six individuals at the day of injection.

			First injection period (25 March –7 July 2009)					Second injection period (7 October –12 December 2009)					Third injection period (12 April –7 June 2010)					
Individual	Sex	Day of birth	Ca	T	Ca	T	Ca	Ca	T	Ca	T	Ca	Ca	T	Ca	Ca	T	Age at death (days)
79618	male	11 May 2009	0	14	28	42	56											69
79756	female	05 April 2009	0	14	28	42	56											90
79615	male	12 May 2009	0	14	28	42	56	147	161	175	196	210						327
79617	female	12 May 2009	0	14	28	42	56	147	161	175	196	210						327
79675	male	25 March 2009	0	14	28	42	56	195	209	223	244	258	384	398	412	426	440	479
79768	female	01 April 2009	6	20	34	48	62	188	202	216	237	251	377	391	405	419	433	472

(Ca = calcein injection; T = oxytetracycline injection).

**Figure 1:**
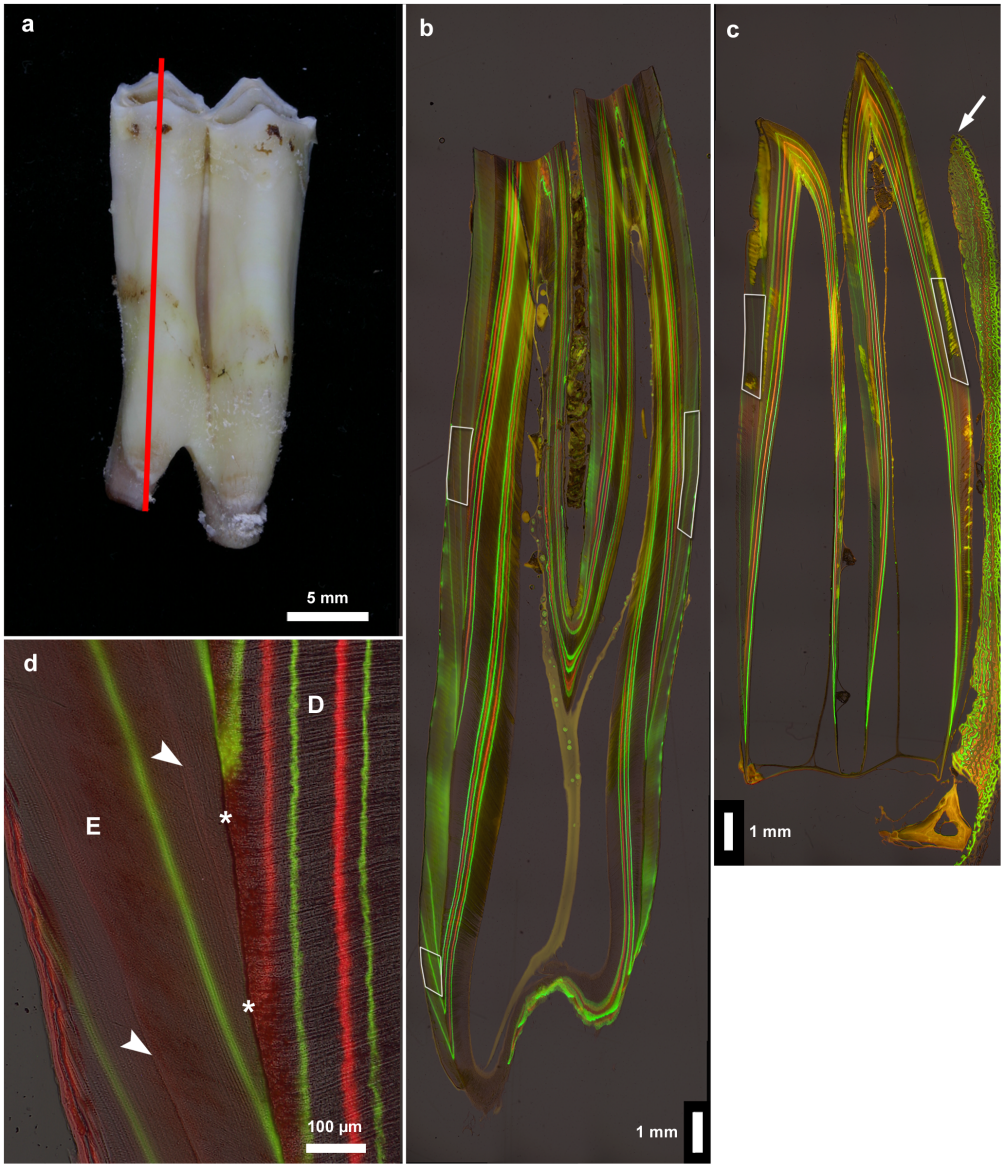
Sectioning plane of tooth and fluorescent labels in enamel and dentin. **a**. Left M_1_ exhibiting cuspal wear, buccal view. The red line indicates the location of the sectioning plane running through the highest points of the mesial cusps. **b**. Micrograph of a bucco-lingual ground section (section plane indicated by red line in Figure 1a) of the worn left M_1_ showing fluorescent labels (green = calcein; red = oxytetracycline) from the first and second injection periods in enamel and dentin. Age at death 327 days. Crown formation is completed and root formation has commenced. White frames indicate areas where measurements of enamel apposition rate were performed. Buccal to the left. **c.** Micrograph of a bucco-lingual ground section of an unworn M_1_ (mesial lobe) that has just erupted beyond the alveolar crest (arrow). Age at death 69 days. Fluorescent labels (green = calcein; red = oxytetracycline) from the first injection period are visible. Crown elongation is still ongoing and fusion of the mineralization fronts in the floor of the infundibulum has not yet occurred. White frames indicate areas where measurements of enamel apposition rate were performed. Buccal to the left. **d**. Detail of bucco-cervical crown area of the tooth shown in Figure 1b. In the dentin (D), both calcein (green) and oxytetracycline (red) labels are clearly visible. In the enamel (E), only the calcein labels are clearly visible, while the oxytetracycline labels (arrowheads) are only faintly visible. Asterisks mark the EDJ.

After each injection period two animals were slaughtered. Their heads were removed, defleshed and macerated using a digestive solution (Enzyrim OSA, Bauer, Switzerland). The skulls were then defatted and bleached using Supralan UF (Bauer, Switzerland) and sodium-tetraborate (Lach-Ner, Czech Republic). Mandibles and crania were photographed and X-rayed prior to removal of the first molars. The teeth were embedded in epoxy resin (Biodur E12, Biodur products, Heidelberg, Germany) and subsequently sectioned axiobuccolingually through the highest point of the anterior (mesial) and posterior (distal) cusps (Figure 1a) using a rotary saw with a water-cooled diamond-coated blade (Woco 50, Conrad Apparatebau, Clausthal-Zellerfeld, Germany). For backscattered electron (BSE) imaging in the SEM, the cut surfaces of the blocks were first smoothed with silicon carbide sandpaper (grits 600 to 2,400). This was followed by polishing on a motorized rotor polisher (Labopol-5, Struers, Copenhagen, Denmark) with diamond suspension of 3 µm particle diameter (DiaPro Dac3, Struers), and a final polishing step with a colloidal silica suspension (OP-S, Struers). BSE imaging of the (uncoated) polished surfaces was performed with an FEI Quanta 600 FEG ESEM that uses high resolution Schottky field emission and was operated in a low-vacuum mode at an accelerating voltage of 20 kV. As we did not use standards for calibration, grey-level differences in the images must be considered a qualitative measure of variation in mineral content of the dental hard tissues. Following acquisition of BSE-images of the polished surfaces, selected specimen were etched for 5 s with 34% (v/v) phosphoric acid to enhance the visibility of the enamel structure. The specimens were then thoroughly rinsed, air dried and again viewed in the SEM.

For light microscopy, tooth blocks were mounted with their polished sides down on glass slides using the epoxy resin as glue. The mounted blocks were sectioned to a thickness of about 1 mm and then ground and polished to a final thickness of about 50 µm. The sections were viewed and photographed in transmitted light using either an Axioskop 2 Plus microscope (Zeiss, Jena, Germany) equipped with a digital camera or a Biozero 8000 inverted digital fluorescence microscope (Keyence, Osaka, Japan). Fluorescence was recorded with specific filter sets to detect calcein (excitation filter (ex) 470/40 nm band pass; dichroic mirror (dm) 495 nm; emission filter (em) 535/50 nm band pass) and oxytetracycline labels (ex 390/40 nm; dm 452 nm; em 562/40 nm) in the ground sections. Emitted fluorescence light was green in both cases. Therefore, images obtained with the oxytetracycline-specific filter set were color converted from green to bright red. For microscopic analyses, identical areas of the sections were photographed with different lighting (plain transmitted light, calcein fluorescence, tetracycline fluorescence) and overlay images from the three recording channels were produced using either Adobe Photoshop (Adobe, San Jose, USA) or the Fiji freeware package (http://fiji.sc) with a stitching plugin [47].

Daily enamel apposition rates were determined in buccal and lingual lateral enamel and in buccal cervical enamel of the mesial (anterior) and distal (posterior) tooth lobes. The crown areas where measurements were performed are indicated in Figures 1b,c. In each of these three locations, the enamel layer was divided into three zones (inner, central and outer third of the enamel). Daily apposition rates (µm/day) were determined separately in each zone along the reconstructed course of the enamel prisms as the distance between two consecutive fluorescent labels divided by the number of days between the respective fluorochrome injections. All measurements were performed on images opened in Fiji, considering the midpoints of the respective labels as reference points. The duration of the secretory lifespan of single ameloblasts was calculated based on the number of fluorescent bands that were passed by the reconstructed course of an enamel prism. As starting point we used the intersection of a fluorescent label with the EDJ. The endpoint of a reconstructed prism course at the OES was normally located between the intersection points of two labels with the OES. Therefore, we measured the distances between the endpoint of the respective prism course at the OES and the endpoints of the cuspally and cervically located fluorochrome labels from the same injection period at the OES. We then calculated the number of days that elapsed between the formation of the outermost fluorescent label crossed by the prism course and the termination of the enamel prism at the OES based on the distances between this point and the endpoints of the two labels at the OES. This means that if a prism reached the enamel surface exactly at the midpoint between two labels that were produced by injections 14 days apart, we added 7 days to the number of days calculated based on the number of fluorescent labels crossed by this prism. Daily enamel extension rates (µm/day) were determined in buccal and lingual enamel of the mesial and distal tooth lobes by measuring the distance between the intersection points of two successive fluorescent bands with the EDJ and dividing this value by the number of days between the fluorochrome injections that produced these labels. The lengths of the secretory fronts were determined by measuring of fluorescent labels whose extension was not affected by attrition of the enamel.

## Results

In the youngest individual studied (age at death 69 days), the cusp tips of the M_1_ were still unworn, while crown extension was ongoing and fusion of the mineralization fronts of the buccal and lingual cusps in the floor of the infundibulum of this tooth had not yet occurred (Figure 1c). In the second youngest individual (age at death 90 days), the mesial cusp tips of the M_1_ exhibited initial enamel wear, while the distal crown tips were not yet worn and crown elongation was still ongoing. By contrast, in an individual with an age at death of 327 days, the crowns of the mandibular first molars already exhibited marked wear and concomitant loss of cuspal enamel(Figure 1b). Crown formation was complete and root formation had already commenced at this age. The fact that cuspal wear starts prior to completion of crown formation excludes the possibility of studying fully formed but still unworn crowns of sheep mandibular first molars.

**Figure 2:**
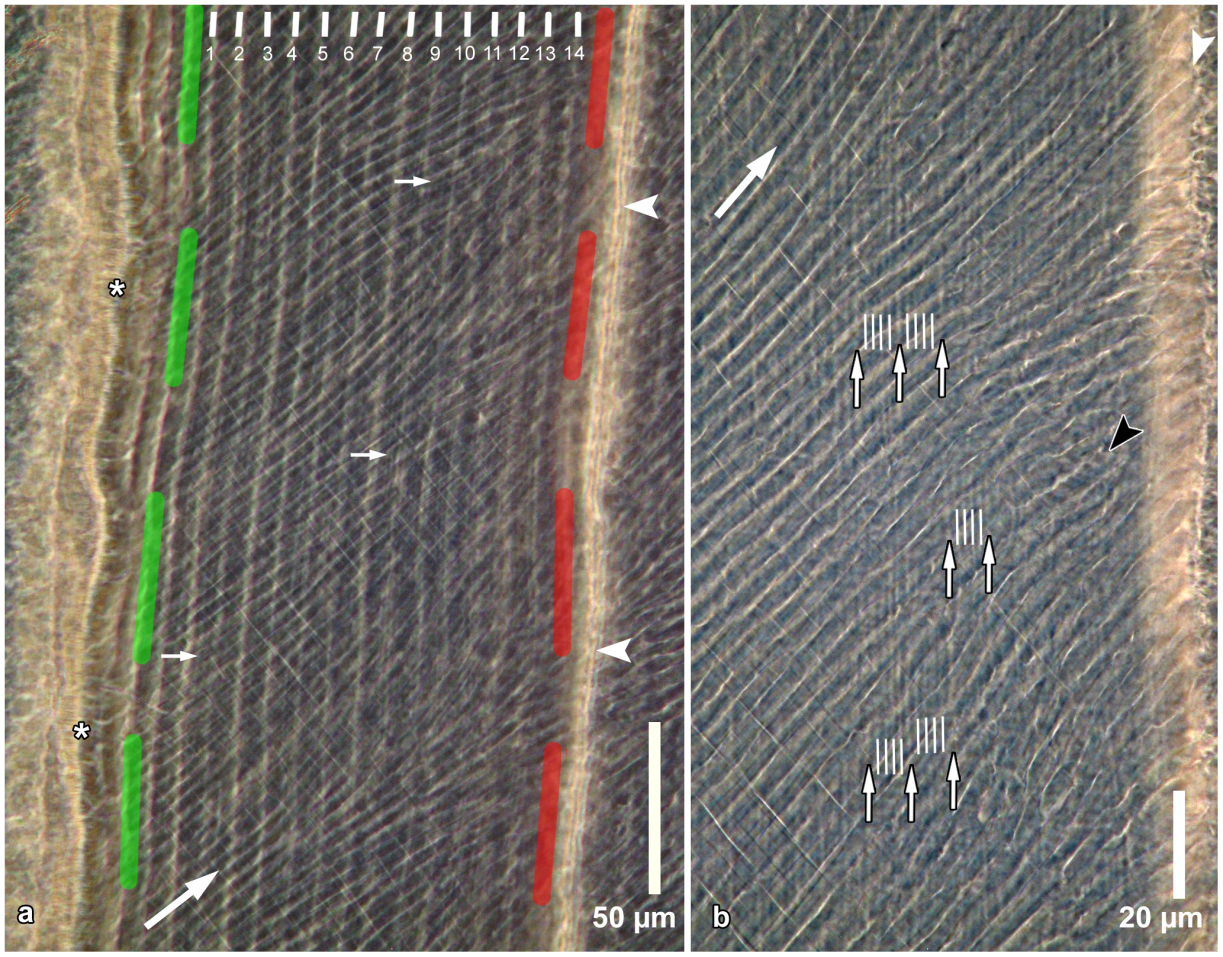
Incremental markings in inner enamel as seen in a ground section. **a.** Enamel near the EDJ (asterisks); the depicted enamel portion is located between a calcein band (indicated by the green dashed line) and an oxytetracycline band (indicated by the red dashed line) from injections given 14 days apart. Thirteen (daily) growth increments are located between fourteen laminations (indicated by white lines). In addition, approximately half a growth increment each is located between the calcein label and lamination number 1 and between lamination number 14 and the oxytetracycline label. Arrowheads indicate the position of a Wilson band caused by the oxytetracycline injection. Large arrow indicates overall prism direction; small arrows point to finer incremental markings between successive laminations. Cuspal to top. Section viewed in transmitted light with phase contrast. **b.** Detail of enamel shown in Figure 2a. Five sub-daily growth increments separated by four sub-daily markings (indicated by white lines) are present between successive laminations (small arrows). Large arrow indicates overall prism direction. White arrowhead indicates Wilson band; black arrowhead indicates a prism showing sub-daily markings. Cuspal to top. Section viewed in transmitted light with phase contrast.

In the dentin, both fluorochromes produced distinct fluorescent labels, while in the enamel only the calcein bands were clearly visible (Figures 1b–d). In contrast, the oxytetracycline injections produced only faintly visible labels in the enamel (Figure 1d). However, the effects of the oxytetracycline injections were clearly detectable in the enamel since they had caused defects (of varying intensity) in the enamel microstructure indicative of an impairment of secretory ameloblast function. In consequence, an accentuated incremental line (Wilson band) with a disrupted enamel structure, which extended from the EDJ to the OES, was located immediately external to the oxytetracycline label in the enamel (Figures 1d, 2a). While in the M_1_s of the oldest individuals (ages at death 472 and 479 days, respectively) all fluorochrome injections had produced labels in the dentin, in the enamel of these teeth only the injections from the first two injection periods were represented by fluorescent labels. This indicates that enamel formation in the mandibular first molars had already been completed prior to the start of the third injection period.

**Figure 3:**
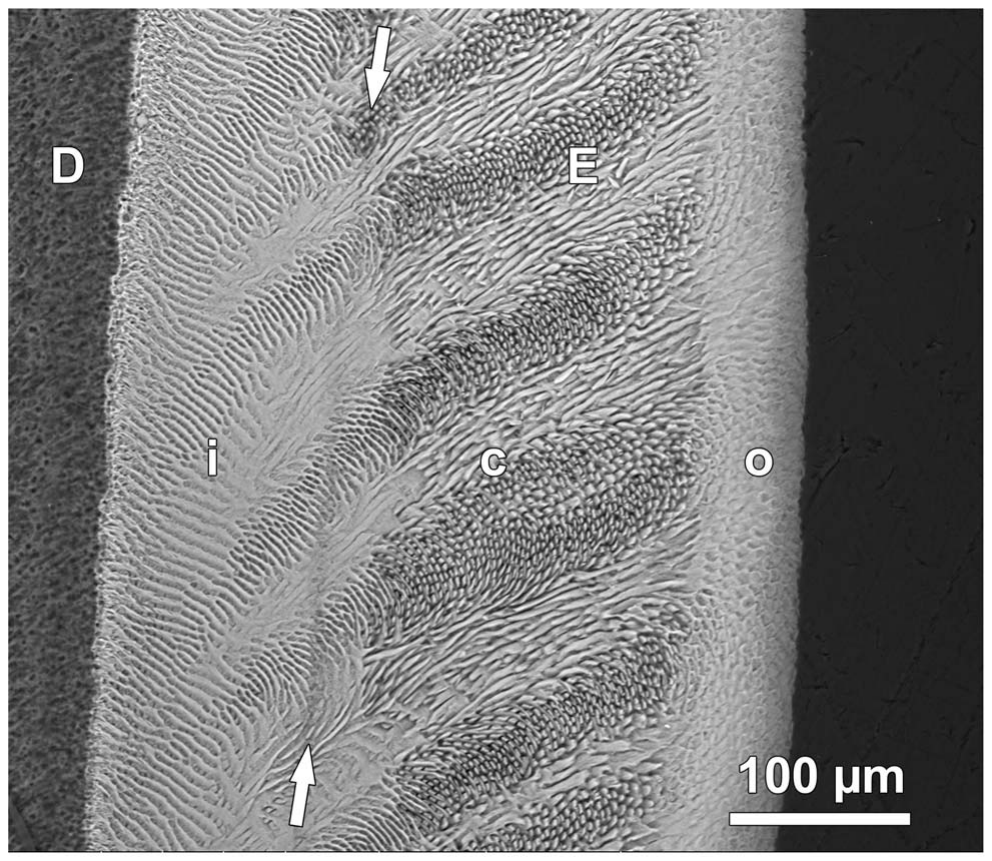
BSE image of etched section through lingual enamel. The enamel (E) can be divided into three zones. The inner zone (i), adjacent to the dentin (D), is characterized by prominent interrow sheets of interprismatic enamel. The central zone (c) shows a typical Hunter-Schreger pattern with alternating bands of more or less longitudinally (parazones) and transversely cut (diazones) prisms. The outer enamel zone (o) shows a much less pronounced variation in prism orientation. Arrows mark the position of a Wilson band with a disrupted enamel structure resulting from oxytetracycline injection. Cuspal to top.

Both in the light microscope and the SEM, the incremental markings were most clearly visible in zones in which the enamel had not yet fully completed maturation. In ground sections viewed in transmitted light, regular incremental markings were discernible that were especially prominent in inner enamel (Figures 2a,b) and in the enamel close to the OES. In central enamel, characterized by Hunter-Schreger bands (Figure 3), the incremental markings were either not or only faintly discernible. As a consequence, individual regular incremental markings could, in contrast to accentuated markings, not be followed throughout the full thickness of the enamel. When viewed in transmitted light, the incremental markings appeared as thin bright lines that separated broader and darker enamel stretches of nearly constant width. The markings followed a course parallel to that of the fluorescent labels indicating that they likewise denoted consecutive positions of the enamel forming front.

In the inner enamel, where incremental markings (lines) were most clearly visible, their number between two consecutive fluorescent labels corresponded to the number of days between the respective fluorochrome injections (Figure 2a). The incremental markings were thus of a daily nature. Based on their structural characteristics and periodicity, these markings were classified as laminations. In our interpretation, each pair of a broad enamel band and the peripherally adjacent lamination represents a 24 h increase in enamel thickness or, in other words, a daily growth increment. In Figure 2a thirteen growth increments are located between the 14 laminations. In addition, approximately half a growth increment each is located between the calcein label (indicated by the green line) and lamination number 1 and between lamination number 14 and the oxytetracycline label (indicated by the red line). These half growth increments represent approximately 12 hour periods of enamel formation that either followed (calcein) or preceded (oxytetracycline) the respective fluorochrome injection. Thus, in sum, 14 growth increments are present between the fluorescent labels that mark a 14 day interval. The fact that the Wilson band was located external to the oxytetracyline label indicates a certain delay between label uptake into the forming enamel and the formation of a structurally disrupted enamel caused by a functional impairment of the secretory ameloblasts.

The course of the laminations was oblique to the overall prism direction (Figures 2a,b). In the ground sections, in places additional incremental markings were discernible between successive laminations (Figure 2a,b), thereby indicating that the former represented sub-daily markings denoting an ultradian periodicity. At higher magnifications, four sub-daily incremental markings and five sub-daily growth increments were recorded between consecutive laminations (Figure 2b). Regular incremental markings with a periodicity of more than one day, i.e. supra-daily markings denoting an infradian rhythm of secretory ameloblast activity, were not discernible in the ground sections. In chronobiology, rhythms with a cycle length of about 24 hours (≥20 to ≤28 hours) are termed circadian while rhythms with a cycle length <20 hours are referred to as ultradian and rhythms with a cycle length >28 hours as infradian [48].

**Figure 4:**
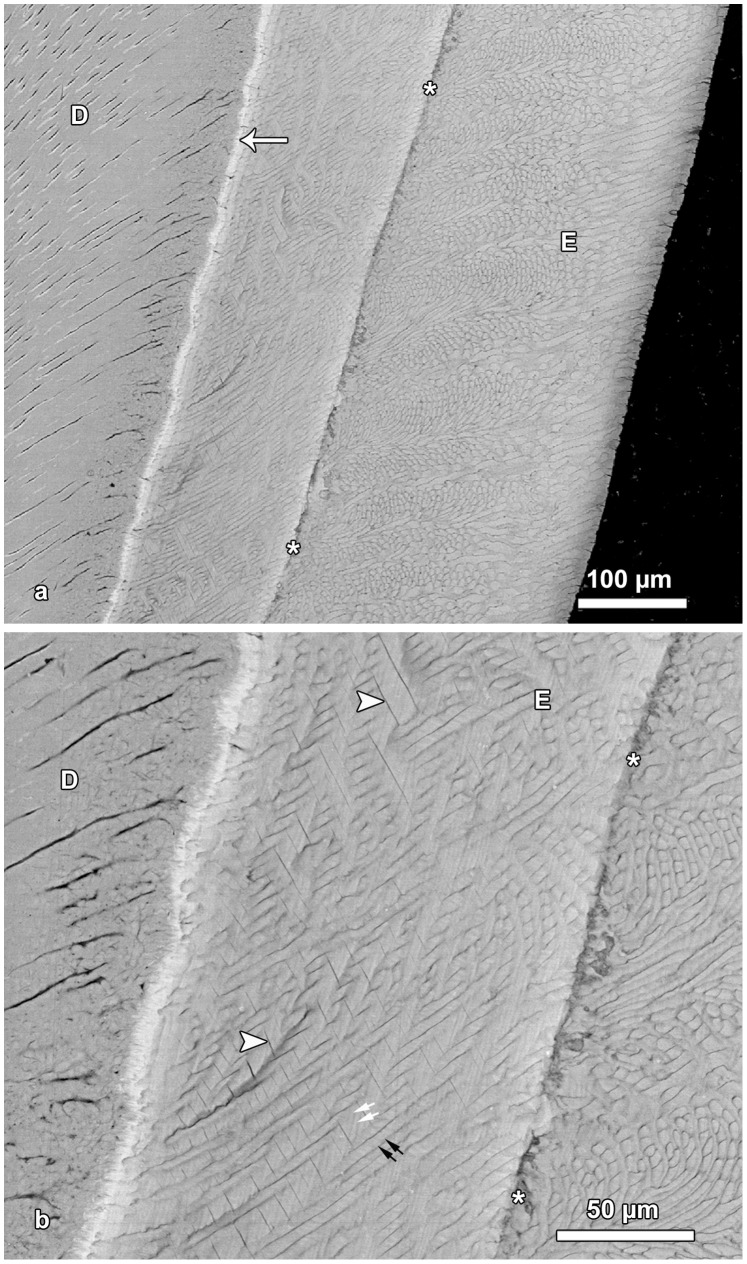
BSE images of hypomature lingual enamel. **a.** Overview of the enamel (E) exhibiting a prominent Wilson band (asterisks) that shows a pronounced hypomineralization. Note bright line (arrow) along the EDJ, indicative of a higher mineral content. D = dentin. Cuspal to top. **b**. Detail of Figure 4a. A disruption of enamel microstructure is visible along the Wilson band (asterisks). Note presence of fine incremental markings in the interprismatic enamel (white arrows) and in the enamel prisms (black arrows). Arrowheads mark fine clefts in the enamel (E); D = dentin.

**Figure 5:**
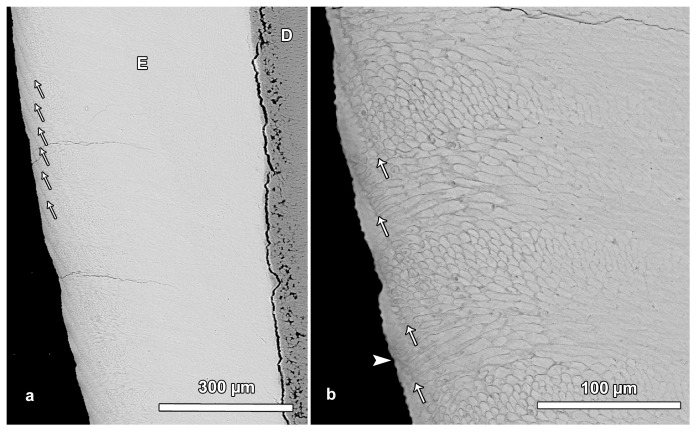
BSE images illustrating laminations in outer enamel. **a**. Regularly spaced laminations (arrows) are visible in the outer portion of the enamel (E). In contrast, no laminations are discernible in central and inner enamel. D = dentin. Cuspal to top. **b**. Detail of Figure 5a. Laminations (arrows) in outer enamel. Note that the outcrop of a lamination on the OES is mostly associated with the presence of a small trough (furrow) on the surface. However, single laminations reach the enamel surface without producing a trough (arrowhead). Cuspal to top.

**Figure 6:**
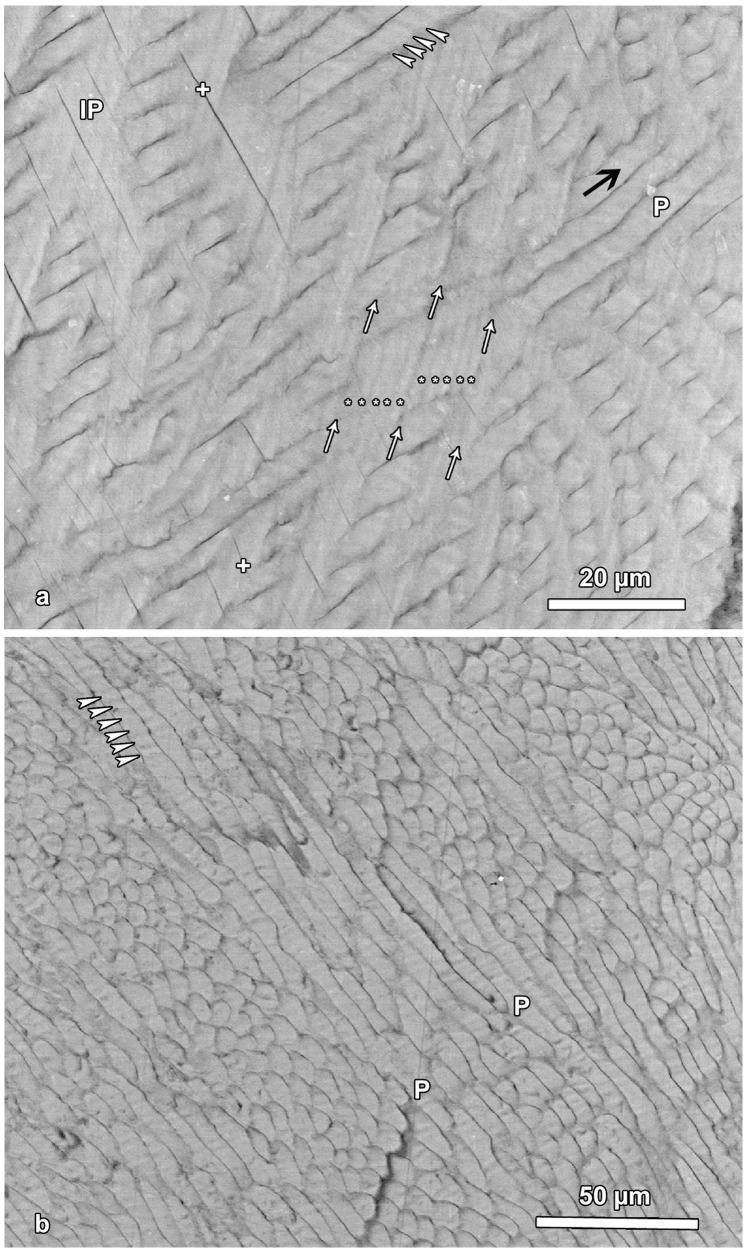
BSE images showing incremental markings in inner (a) and central (b) lingual enamel. **a**. White arrows indicate the course of three laminations visible in interprismatic enamel (IP). Between two successive laminations, five sub-daily increments (asterisks) separated by four darker lines are discernible. Arrowheads mark sub-daily increments in an enamel prism (P). Black arrow indicates overall prism direction. Note occurrence of clefts (crosses) in interprismatic enamel. Cuspal to top. **b**. Presence of sub-daily incremental markings (arrowheads) in longitudinally sectioned prisms (P) of central enamel. Cuspal to top.

Based on BSE images of etched specimens, the enamel could be divided into three zones of unequal width that differed in structure (Figure 3). The inner enamel was characterized by prominent interrow sheets of interprismatic enamel. The central enamel showed a distinct Hunter-Schreger pattern with alternating bands of more or less longitudinally (parazones) and transversely (diazones) cut prisms. In the outer enamel, the variation in prism orientation was much less pronounced than in central enamel (Figures 3, 4a, 5b, 6a,b). The Wilson bands caused by the oxytetracycline injections were typically clearly visible in the BSE images as hypomineralized (dark) bands with a disrupted enamel structure (Figures 3, 4a,b). In comparison to light microscopic images, laminations were less prominent in BSE images of the inner enamel zone (Figures 3, 4a,b). They were, however, frequently visible in BSE images of the outer enamel, where they followed a course almost perpendicular to that of the prisms (Figures 5a,b). The outcrop of a lamination at the OES was mostly, but not always, associated with a shallow furrow on the surface resembling a perikyma groove (Figure 5b). In inner enamel, BSE imaging revealed the presence of incremental markings with a closer spacing than that of the laminations (Figures 4b, 6a,b). Such closely spaced (sub-daily) markings were present in both prismatic and interprismatic enamel and showed an alternation of narrow and darker (less mineralized) and broader and brighter (more mineralized) bands (Figures 6a,b).

In the prisms, the sub-daily incremental markings formed an angle of 40 to 80 degrees with the prism long axis (Figures 6a,b). The sub-daily incremental markings in prismatic and interprismatic enamel were oriented at roughly right angles to each other (Figure 6a).

Where laminations were discernible in the BSE-images of the inner enamel zone, presence of five broader and brighter bands separated by four narrow and darker lines were observed between two consecutive laminations (Figure 6a). This suggests that five sub-daily growth increments are present between two consecutive laminations, thereby matching the light microscopic observations. This conclusion is further corroborated by the width of the sub-daily increments. In Figure 6a, showing inner lingual enamel, ten sub-daily growth increments in the interprismatic enamel cover a stretch of approximately 18 µm, i.e., one sub-daily increment has a width of *c.* 1. 8 µm. In figure 2a, the shortest distance between two fluorescent labels encompassing 14 days enamel growth is about 120 µm. Assuming the formation of five sub-daily increments (of 1.8 µm width each) per day, a total of 70 sub-daily increments would cover a stretch of 126 µm, which is quite close to the actually measured distance of *c.* 120 µm.

In central enamel, the sub-daily growth increments in the prisms were clearly visible in the parazones of the Hunter-Schreger-bands, where the prisms were cut more or less longitudinally (Figure 6b). The width of the sub-daily growth increments in the enamel prisms was about 2.2–2.3 µm and thus *c*. 25% greater than in the interprismatic enamel. This corresponds to the fact that the distance between two successive fluorochrome bands measured along the overall prism direction was about 25% higher than the distance between the same labels measured along a line oriented perpendicular to the fluorescent labels. The BSE images also revealed that the sub-daily incremental markings in interprismatic enamel showed the same orientation as the laminations (Figures 4b, 6a). As in the ground sections, also in the BSE images no long period incremental (supra-daily) markings were discernible.

A feature observed in the BSE images of immature enamel were small clefts that separated adjacent sheets of interprismatic enamel (Figures 4b, 6a). In the ground sections these structures were visible as fine lines running from the EDJ in cervical direction (Figures 2a,b).

Based on the established 1-day periodicity of the laminations, daily enamel apposition rates were determined along the reconstructed prism course at different locations in the inner, central and outer third of the enamel layer (Table 2). Overall, daily apposition rates were remarkably high in all locations with a tendency to increase from the inner over the central to the outer third of the enamel layer.

**Table 2:** Daily enamel apposition rates (means ± SD) in inner, central and outer third of the enamel layer in different locations of mandibular first molars of four Soay sheep.

	Lateral enamel, buccal side(mean enamel thickness: 646.6 µm)		Lateral enamel, lingual side (meanenamel thickness: 464.7 µm)		Cervical enamel, buccal side (meanenamel thickness: 615.7 µm)	
	Following calceininjection	Followingoxytetracycline injection	Followingcalcein injection	Followingoxytetracycline injection	Followingcalcein injection	Followingoxytetracycline injection
Inner enamel	15.0±2.27 µm	12.3±1.50 µm (82.0%)	11.6±1.17 µm	10.2±0.63 µm (87.9%)	10.3±1.08 µm	8.0±1.57 µm (77.7%)
Central enamel	16.4±1.76 µm	14.0±1.51 µm (85.4%)	12.7±1.10 µm	9.7±1.53 µm (76.4%)	11.1±1.0 µm	10.1±1.48 µm (91.0%)
Outer enamel	17.0±1.08 µm	14.5±1.47 µm (85.3%)	13.4±0.82 µm	10.1±0.55 µm (75.4%)	13.2±1.50 µm	11.4±3.62 µm (86.4%)

The percentages given in brackets in the columns “following oxytetracycline injection” indicate the amount of the respective enamel matrix secretion in relation to that occurring after calcein injection. Values are averages from measurements on the mesial and distal tooth lobes.

A consistent finding was that daily apposition rates following oxytetracycline injections were lower than those following calcein injections (Table 2). This indicates that, at the given dosage, oxytetracycline had caused a temporary impairment of enamel matrix formation, which is corroborated by the observed disruption of normal enamel structure associated with the oxytetracycline labels (Figures 2, 3, 4a,b). Since no deviation from normal enamel structure occurred in conjunction with the calcein injections, we consider the apposition rates in the enamel following calcein labeling to represent undisturbed enamel matrix secretion. Therefore these values are given below.

Apposition rate was highest in the lateral (imbricational) enamel of the buccal tooth side, with mean values of 15.0 (inner), 16.4 (central), and 17.0 (outer enamel) µm/day. The maximum value recorded for outer enamel was *c.* 20.0 µm/day. For lateral enamel of the lingual tooth side, where the enamel is thinner than buccally, lower mean daily apposition rates were recorded (11.6 in inner, 12.7 in central and 13.4 µm/day in outer enamel). Lower mean daily apposition rates (10.3 in inner, 11.1 in central and 13.2 µm/day in outer enamel) compared to more cuspally located lateral enamel were recorded for cervical enamel of the buccal side, although enamel thickness did not markedly differ between the two locations (Table 2).

The reconstructed duration of the secretory lifespans of single ameloblasts varied between different crown areas. Highest durations were determined with 49 − 53 days in cervical enamel of the buccal tooth side. In more cuspally located (lateral) enamel, the respective values varied between 39 and 46 days on the buccal and 35 and 42 days on the lingual tooth side.

Enamel extension rates recorded for postnatal crown formation in the first molars varied considerably (Table 3). High values were recorded for early postnatal crown growth, while values for later stages of crown formation were much lower. The very high enamel extension rates during early postnatal crown formation resulted in extended enamel secretory fronts (indicated by the lengths of the fluorescent bands) with maximum values of approximately 9,000 µm. Much shorter secretory fronts were reconstructed for more cervical crown portions (Figures 1b,d, Table 4). In three of the four individuals surviving up to day 188, enamel extension on the lingual tooth flank had ceased prior to that day. In contrast, in the fourth individual enamel extension was still ongoing on the lingual tooth flank at day 251. In the buccal enamel of all four individuals, enamel extension was still ongoing at day 258, reflecting the fact that in mandibular first molars of sheep buccal enamel extends further apically than lingual enamel (Figure 1b).

**Table 3:** Daily enamel extension rates in buccal and lingual enamel of Soay sheep mandibular first molars.

Period (postnatal age in days)	Enamel extension rate (µm/day), buccal side			Enamel extension rate (µm/day), lingual side		
	mean	range	N	Mean	range	N
0–14 (6–20)*	177.6	147.7–219.0	6	217.3	148.2–274.5	6
14–28 (20–34)*	168.4	148.1–199.1	6	189.8	160.4–260.1	6
28–42 (34–48)*	180.0	136.8–225.5	6	181.2	129.8–217.5	6
42–56 (48–62)*	131.7	106.6–166.4	6	126.0	106.5–176.9	6
56–90	109.9		1	111.2		1
147–161	40.4	36.9–43.9	2			
161–175	35.5	32.0–39.0	2			
175–196	35.0	34.6–35.4	2			
188–210	33.2	29.5–35.4	2	30.5		1
202–223	27.1	26.0–28.3	2	25.0		1
216–244	28.0	26.1–29.9	2			
237–258	29.3	27.9–30.8	2	19.5		1

N = number of individuals from which data were recorded in the given postnatal age period. The overlap in the last five periods result from the different birth dates of the respective individuals.

* Data in parenthesis refer to specimen #79768 in which injection started at day 6 after birth.

**Table 4:** Length of fluorescent band/secretory front in buccal and lingual enamel of Soay sheep mandibular first molars.

Postnatal agein days	Length of fluorescence band/secretoryfront (µm), buccal side			Length of fluorescence band/secretoryfront (µm), lingual side		
	mean	range	N	Mean	range	N
0	5318	3763–6873	2	6619	5427–7604	4
14	6491	4658–7913	3	7048	5923–7955	4
28	7416	6782–8262	4	7738	6852–8318	4
34				7239		1
42	7797	6517–8747	4	7745	5567–8903	5
48	7444		1	7735		1
56	7718	6349–8571	5	7038	5245–9034	5
62	7649		1	7173		1
147	3263	2931–3595	2	1777	1717–1838	2
161	2886	2762–3010	2			
175	2629	2392–2866	2			
188	3710		1	1853		1
195	3169		1			
196	2233	2004–2462	2			
202	3156		1	1953		1
209	2832		1			
210	2029	1785–2274	2			
216	2743		1	1618		1
223	2572		1			
237	1994		1			
244	2432		1			
251	1850		1	1260		1
258	2223		1			

N = number of individuals for which data were recorded at the respective postnatal age. Values are averages from measurements on the mesial and distal lobes of individual teeth.

## Discussion

This is the first experimental study analyzing the periodicity of regular incremental markings in sheep enamel. Our findings clearly revealed the daily nature of the laminations in sheep enamel. This result is in accordance with that of a fluorochrome labeling study in the enamel of another ruminant species, the sika deer (*Cervus nippon*), likewise reporting a daily nature of incremental lines (laminations) [34].

In a series of experiments using lead acetate or sodium fluoride for vital labeling of forming enamel, the occurrence of laminations (called ‘Parallelstreifen’in these studies) with a 1-day periodicity was found in the enamel of dogs, pigs, rabbits and macaques [43], [45]. Interestingly, only in macaque enamel the authors [45] found that, in addition to the ‘Parallelstreifen’ with a 1-day periodicity, incremental lines with a longer (5-day) periodicity were visible, which they regarded as equivalents of the striae of Retzius in human enamel. The daily nature of laminations (termed cross striations in one of the studies [22]) was later corroborated in vitally labeled enamel of *Macaca nemestrina* [22], [23]. Circumstantial evidence for the daily nature of laminations was furthermore provided for the enamel of extinct and extant rhinoceros and caprine species, with reported enamel apposition rates ranging between approximately 10 µm/day in rhinoceros enamel and approximately 11–16 µm/day in modern caprine enamel [16], [35], [36]. While these apposition rates correspond well with those recorded in the present study, the laminations (again referred to as cross striations) identified as daily incremental markings in the inner portion of horse enamel [33] exhibited a spacing of only *c.* 5 µm. This value seems quite low compared to the values recorded for caprine enamel and might indicate a misidentification of the respective incremental markings in the horse enamel. In this context it has to be cautioned that in studies of non-primate enamel that do not use labeling, a correct distinction between daily and sub-daily incremental markings may be difficult.

Our study showed that the laminations in sheep enamel exhibit the same orientation as the sub-daily incremental markings in the interprismatic enamel. This suggests that the formation of laminations occurs at the interprismatic growth sites that are related to the proximal portions of the Tomes’ processes. The topographic relationship of laminations with the forming front of interprismatic enamel explains why in ground sections of sheep teeth, laminations appear especially prominent in inner enamel, since here the interrow sheets of interprismatic enamel are the dominant structural element [49]. Thus, interprismatic enamel accounts for more than 50% of the total volume fraction in the inner portion of sheep enamel, whereas in central and outer enamel, the volume fractions of interprismatic enamel are only about 20% [49].

The observation that in human teeth laminations occur preferentially in aprismatic surface enamel [3], [30]–[32] is consistent with the view that they are formed at the interprismatic growth sites. Aprismatic enamel is formed at the flat secretory surface that remains at the base of the Tomes’ process following regression of its distal (prism forming) portion [1], [12], [14], [15], [50]. The crystals formed in the matrix secreted at this flat surface show the same orientation as those of interprismatic enamel. In consequence, crystal orientation in zones of aprismatic enamel formed due to a transient impairment of enamel matrix formation (and the related regression of the distal portion of the Tomes’ process) corresponds to that of interprismatic enamel [31], [32], [51].

Our findings corroborate the view [23] that laminations have a topographically separate origin from prism cross-striations. Based on the topography of the enamel secretory front it can be deduced that the growth increments of interprismatic enamel reflect the coordinated action of a large number of ameloblasts aligned along the respective secretory front [4]. The difference in the interpretation of laminations notwithstanding, we agree with the statement [16] that using laminations to calculate crown formation times in ungulate teeth should be as precise as counting prism cross-striations in primate teeth.

The present study provides clear evidence for the presence also of regular sub-daily incremental markings in enamel prisms and interprismatic enamel of sheep teeth. Our findings indicate the presence of five sub-daily growth increments between successive laminations. The nearly perpendicular orientation of the sub-daily incremental markings in enamel prisms and interprismatic enamel reflects the orientation of the respective secretory surfaces of the Tomes’ process [1], [50], [52].

Occurrence of sub-daily incremental markings has previously been reported in mammalian dental hard tissues. Smith [23] described the presence of 2 to 3 ultradian lines between successive prism cross striations in the enamel of *Macaca nemestrina*, while Rosenberg and Simmons [53] recorded 2 to 3 ultradian lines between daily dentin increments in rabbit incisors. In rats, a temporal or permanent obliteration of the suprachiasmatic nucleus resulted in a loss of the circadian rhythm during dentin formation and the appearance of an ultradian rhythm with an approximate 12 hour periodicity [54]. This suggests that the suprachiasmatic nucleus is involved in the control of the circadian rhythm of dental hard tissue formation. However, there is also evidence for a local control of the rhythmic formation of enamel and dentin as it has been demonstrated that intracellular clock genes with a circadian oscillatory expression pattern are involved in dental hard tissue formation [11], [25], [26], [55].

BSE imaging of the sub-daily enamel markings revealed a periodic variation in the degree of mineralization between the alternating broader (more mineralized) bands and the narrower (less mineralized) lines. With respect to daily prism cross striations, it has previously been hypothesized that they reflect regular physiologic changes in the acid-base-balance of the organism [1], [45]. It was argued that these changes may affect ameloblast metabolism in a way leading to a rhythmic shift of the normal ratio between calcium and phosphate on the one side and magnesium and carbonate on the other side in the formed mineral [1]. A shift towards a higher percentage of magnesium and carbonate would cause a reduction in the density (mean atomic number) of the enamel mineral that could be detected by BSE-imaging in the SEM.

In keeping with the findings of Lacruz et al. [11], we assume that the formation of laminations in sheep enamel occurs when matrix secretion is at its nadir during night-time. Occurrence of the sub-daily growth increments points to the existence of an additional oscillation of ameloblast activity with a periodicity of *c*. 5 hours. The fact that incremental markings are best visible in enamel that has not yet achieved full maturation may point to the fact that the differences in mineral density and composition within the enamel become less pronounced with ongoing maturation. This would also explain the enhanced visibility of incremental markings in fluorotic enamel that is characterized by varying degrees of hypomineralization due to an impairment of the maturation process [56]–[58].

The recorded enamel apposition rates in the first molars of Soay sheep indicate a remarkably high secretory activity of the ameloblasts. Our results are in accordance with previously reported daily enamel secretion rates in various mammal species. Iinuma et al. [34] found a mean daily apposition rate of 11.2 µm (±2.5 µm SD) in inner enamel of mandibular first molars of sika deer. For molars of extant sheep, an overall mean daily enamel apposition rate of 11.6 µm (±1.4 µm SD) was reported, while in molars of the fossil bovid *Myotragus balearicus* the respective value was 9.3 µm (±2.1 µm SD) [35]. High apposition rates with mean values of around 16 µm/day have previously been reported for the enamel of rodents and dogs [44]. Enamel apposition rates reported for hominoid enamel are generally lower, ranging between a lower limit of 2–3 µm/day and an upper limit of 6–7 µm/day [17], with human enamel exhibiting mean daily apposition rates around 3.5 µm [3].

Enamel extension rates in the Soay sheep molars were high in upper crown areas (maximum means of 180 µm/day in buccal and 217 µm/day in lingual enamel) and declined markedly in more cervical crown portions. This indicates a very rapid recruitment of new secretory ameloblasts in the more cuspal crown areas, leading to the observed extended secretory fronts. Crown elongation was (mostly) completed prior to day 188 *post partum* in lingual enamel, while this process was still ongoing at day 258 in buccal enamel. Previous studies reported enamel formation times of around 251 days for lingual [35] and about 300 days for buccal [36] enamel of individual lobes of caprine mandibular first molars. Enamel extension rate in the sheep molars was markedly lower in cervical compared to more cuspal crown portions. In addition, slightly lower daily apposition rates were recorded in cervical enamel. In consequence, formation of the cervical third of the molar crown covers at least the same time span as that of the upper and middle thirds. This fact has to be considered when reconstructing stress periods during tooth formation based on the localization of hypoplastic defects of the enamel along the vertical tooth axis [36].

The laminations observed in sheep enamel follow a course corresponding to that of the striae of Retzius in human enamel. However, the growth increment between two successive laminations in sheep enamel represents apposition over a 1-day period, whereas in human teeth the growth increment between two successive striae of Retzius represents a period of several days. The sub-daily increments in the prisms of sheep enamel resemble the prism cross striations in human enamel; however, in sheep enamel these markings reflect an approximately 5 h periodicity, not a 24 h periodicity as in the case of prism cross striations in human enamel. Incremental markings with an supra-daily periodicity were not discernible in the sheep enamel. This could either mean that in sheep enamel such supra-daily markings are not present or that they cannot be distinguished from daily markings with the methods applied in this study. Whether or not the laminations present in sheep enamel can be considered striae of Retzius with a periodicity of 1 day is a matter of definition. In this context it should be considered that according to our findings laminations are structurally related to the interprismatic enamel.

Interestingly, a stria of Retzius repeat interval of 5 days was previously reported for enamel of sheep from a modern breed [59]. Two possible explanations exist for the conflicting findings regarding the incremental markings in sheep enamel between this and our study. First, different sheep breeds could vary with respect to this trait. Second, in the modern breed, daily laminations may have erroneously been interpreted as striae of Retzius with a repeat interval of five days and the sub-daily markings as daily cross striations.

The results of the present study show that a simple transfer of the framework that has been developed for the description and analysis of incremental markings in human and other primate enamel to the enamel of ungulates is likely to produce a misinterpretation of the incremental markings present in the latter. Such a misinterpretation will always lead to an overestimation of crown formation times in ungulates. This is probably the cause for the previously reported crown formation period of 1035 days in first molars of *Gazella granti* [60]. Such a long crown formation period seems highly unlikely, both in comparison with other medium-sized ruminants [35], [36], [61]–[63] and in the light of isotope data for the enamel of gazelle molars [64].

In conclusion, the present study provided experimental evidence for the presence of daily and sub-daily incremental markings in sheep enamel, while supra-daily markings reflecting an infradian periodicity of secretory ameloblast activity were not discernible. Our findings are in accordance with previous observations on the enamel of dogs, pigs, rodents, deer and bovids [34]–[36], [43]–[45]. Why primate enamel apparently differs from the enamel of other mammalian orders in exhibiting prominent incremental enamel markings with a supra-daily periodicity is unknown. However, the fact that enamel apposition rates in primates are much lower compared to species in which supra-daily enamel markings are not discernible, may indicate that the rate of enamel matrix secretion plays a decisive role in this context.
